# Global, regional, and national burden of anxiety disorders during the perimenopause (1990–2021) and projections to 2035

**DOI:** 10.1186/s12905-025-03547-z

**Published:** 2025-01-07

**Authors:** Ying Zhang, Ting-Ting Hu, Yong-Ran Cheng, Zhi-Fen Zhang, Jun Su

**Affiliations:** 1 Department of Psychosomatic Diseases (II), Affiliated Mental Health Center & Hangzhou Seventh People’s Hospital, Zhejiang University School of Medicine, Hangzhou, 310013 China; 2 Laboratory Department, Hangzhou Women’s Hospital (Hangzhou Maternity and Child Health Care Hospital), Hangzhou, 310008 China; 3 School of Public Health, Hangzhou Medical College, Hangzhou, 311300 China; 4 Gynecological Endocrinology Department, Hangzhou Women’s Hospital (Hangzhou Maternity and Child Health Care Hospital), Hangzhou, 310008 China; 5 School of Public Health, Hangzhou Normal University, Hangzhou, 311121 China; 6 Intensive Care Medicine Department, Hangzhou Women’s Hospital (Hangzhou Maternity and Child Health Care Hospital), Hangzhou, 310008 China

**Keywords:** Perimenopause, Anxiety disorders, GBD, Disability-adjusted life years, Average percent change

## Abstract

**Purpose:**

Perimenopause is associated with an increased risk of anxiety disorders, largely due to hormonal changes affecting the body’s regulatory feedback mechanisms. This study aims to provide a comprehensive analysis of the global burden of anxiety disorders among perimenopausal women.

**Methods:**

Data from the 2021 Global Burden of Disease (GBD) database were utilized to assess disability-adjusted life years associated with anxiety disorders linked to perimenopause. We calculated trends using the estimated average percent change, and future projections were made using the Bayesian age–period–cohort model to estimate disability-adjusted life year trends for anxiety disorders from 2022 to 2035.

**Results:**

Between 1990 and 2021, the global age-standardized disability-adjusted life year rate for anxiety disorders among perimenopausal women increased from 625.51 (95% uncertainty interval: 429.1–891.09) to 677.15 (95% uncertainty interval: 469.45–952.72), indicating a rising trend with an estimated average percent change of 0.081 (95% confidence interval: 0.0043–0.143). Regional differences were noted, with anxiety disorder burdens varying across areas with different sociodemographic index levels. Projections suggest that by 2035, the global burden of anxiety disorders in perimenopausal women will rise to 1,180.43 per 100,000, a 40.67% increase compared with 2021 levels.

**Conclusion:**

The burden of anxiety disorders during perimenopause is a growing global concern, with a significant increase anticipated in the coming years. Targeted prevention and intervention strategies are urgently needed to mitigate this rising burden and improve mental health outcomes during perimenopause.

## Introduction

Perimenopause refers to the transitional period from the onset of ovarian dysfunction to menopause, continuing until one year after a woman’s last menstrual period [1]. As a significant turning point in a woman’s life, perimenopause is often associated with vascular constriction and changes in the urinary and reproductive systems, which may have a substantial impact on mental health. According to data from the World Health Organization, by 2030, there will be over 1.2 billion menopausal women worldwide [2]. Therefore, exploring the psychological disorders arising in perimenopausal women is of great significance.

Perimenopause is not only a crucial stage in the reproductive cycle, marked by the gradual decline in ovarian hormone secretion, but also represents an important transition from sexual maturity to old age [3–5]. Typically occurring between the ages of 45 and 55 years, the timing and duration of perimenopause vary significantly among women. While some transition within 2–3 years, others may experience hormone fluctuations for up to 10 years. This period is often accompanied by various emotional, cognitive, and physical symptoms, including depression, irritability, hot flashes, night sweats, and memory loss, all of which can negatively affect the quality of life [6, 7].

The changes in gonadal feedback during perimenopause occur in three distinct stages. The first stage, premenopause, is characterized by irregular menstrual cycles and a significant reduction in the number of oocytes. The second stage, menopause, marks the cessation of menstruation and the depletion of ovarian follicles. The third and final stage, known as the postmenopausal period, occurs when ovarian function is completely depleted [8]. During these stages, the lack of estrogen increases the susceptibility to anxiety disorders [9].

As the number of patients with perimenopausal anxiety disorders continues to rise, related diagnostic techniques are also being continuously improved. The earliest method relied on the Blatt-Kuperman Menopausal Index to identify perimenopausal anxiety disorders [10]. However, the overlap between perimenopausal syndrome and depression often presents diagnostic challenges, as new depressive symptoms may confuse with existing conditions, blurring the lines between the two. Clinically, perimenopausal anxiety and depression typically manifest as a persistent feeling of sadness lasting for two weeks or longer, accompanied by symptoms such as difficulty concentrating, excessive self-doubt, panic, and physical discomfort, primarily measured through anxiety self-assessment scales [11, 12]. Currently, there is an urgent global need for greater attention to the prevalence of perimenopausal anxiety and its burden on women’s health, providing an epidemiological basis for the formulation of policies aimed at preventing perimenopausal anxiety disorders. Despite the importance of this issue, a comprehensive analysis of the disease burden caused by anxiety in perimenopausal women has yet to be conducted.

This study aims to quantify the disability burden of anxiety among perimenopausal women and project future trends to inform policy-making and healthcare strategies. The data comes from the Global Burden of Disease Database (GBD), which provides global, regional, and country-specific disease burden information for 204 countries from 1990 to 2021.

## Methods

### Data sources

The data for this study were derived from the GBD 2021 study. The GBD has been systematically estimating mortality and health losses from a wide range of diseases using a standardized methodology since 1990, with re-estimations every 2–3 years [13, 14]. The GBD 2021 study provided estimates for the burden of disease attributable to 286 causes of death, 369 illnesses and injuries, and 87 risk factors across 204 countries and territories [15]. The GBD database provides the advantage of conducting longitudinal and comparative analyses across different population backgrounds. The GBD data is aggregated from a variety of sources, including official government statistics, hospital records, survey data, and scientific literature. These data are cleaned, standardized, and modeled to ensure their quality and comparability. Understanding the diversity and complexity of data sources is fundamental to conducting any analysis. We utilized the GBD Results Tool to extract global data on the population of perimenopausal women from 1990 to 2021. The Global Health Data Exchange, maintained by the Institute for Health Metrics and Evaluation, served as the primary source of this data (https://vizhub.healthdata.org/gbd-results/).

### Definitions

The SDI is a composite indicator of the development status of a country or region, derived from a comprehensive assessment of data such as per capita income, average education level, and fertility rate. Each shelf has a corresponding SDI value, ranging from 0 to 1, with a higher value indicating a better development status of the country. Countries and regions were categorized into five groups based on their sociodemographic index (SDI): high, high-medium, medium, low-medium, and low SDI regions. The SDI is a composite measure used to assess the developmental status of countries or regions.

To ensure the comparability of measurements, the case definitions primarily follow the DSM-IV-TR and ICD-10 standards, as these standards are used in most of the included mental health surveys [16]. The anxiety disorders included are classified as follows: DSM-IV-TR: 300.0-300.3, 208.3, 309.21, 309.81; ICD-10: F40-42, F43.0, F43.1, F93.0-93.2, F93.8.The GBD database provides comprehensive estimates for anxiety disorders, including all subtypes, ensuring comparability across measurements. Case definitions primarily adhere to the Diagnostic and Statistical Manual of Mental Disorders, Fourth Edition, Text Revision or International Classification of Diseases, Tenth Revision standards, as these are the most commonly used criteria in mental health surveys [17]. Since most women experience perimenopause between the ages of 45 and 55 years, individuals in this age range with anxiety disorders were included in the GBD database.

To assess the disease burden, we selected disability-adjusted life years (DALYs) and their corresponding age-standardized rates as key parameters. The data were disaggregated by geographic location and age group. DALYs provide a quantitative measure of the healthy life years lost due to premature death and disability. They are calculated by combining the years lost due to premature death (the gap between actual age at death and the expected life expectancy in a low-mortality population) with the years of life lost due to disability [18].

### Statistical analysis

Age standardization refers to the method of processing demographic data according to a standardized age structure. Its purpose is to eliminate the impact of differences in population age composition and to ensure the comparability of statistical indicators. The age-standardized rate (ASR) per 100,000 population was calculated using the direct method, where the ASR is derived by summing the products of age-specific rates ($\:{a}_{i}$, where $\:i\:$denotes the $\:{i}^{th}$ age class) and the corresponding number of individuals (or weight) ($\:{w}_{i}$) in the same age subgroup $\:i\:$of the chosen reference standard population. This total is then divided by the sum of standard population weights, as follows:

$$ASR=\frac{{\sum\nolimits_{{i=1}}^{A} {{a_i}{w_i}} }}{{\sum\nolimits_{{i=1}}^{A} {{w_i}} }} \times 100000$$

We calculated the age-standardized rate of disability-adjusted life years (ASDRs) and the corresponding 95% uncertainty intervals (UIs) to quantify the burden of anxiety disorders during perimenopause. 95% UIs represent uncertainty in age-specific death rates and DALYs per capita for each country, age, sex, and year. ASRs were standardized to the global age structure, facilitating accurate comparisons between populations across different locations or over time.

To assess trends, the estimated average percent change (EAPC) was calculated for age-standardized rates of anxiety disorders during perimenopause. An increasing trend was identified when the lower bound of the EAPC and its 95% confidence interval (CI) was greater than 0, a decreasing trend when the value was less than 0, and a stable trend when it was equal to 0 [19].

Future trends in DALYs for anxiety disorders during perimenopause (2022–2035) were predicted using the Bayesian age-period-cohort (BAPC) analysis model, with integrated nested Laplace approximation. The BAPC model is used to predict future disability rates. It forecasts future disability rates by fitting past disability data. In the BAPC model, age, period, and cohort effects reflect underlying processes such as changes in risk factors, interventions, or diagnostic practices. Therefore, the BAPC model can provide estimates of future disease burden without predicting the underlying processes, and it is currently well-established in the analysis of the Global Burden of Disease (GBD) database [20].

All statistical analyses were conducted using R (version 4.2.2) and RStudio, while BAPC prediction models were performed using the nordpred (version 1.1), BAPC (version 0.0.36), and integrated nested Laplace approximation (version 22.05.07) packages. The premise of using these statistical packages is to properly handle the data structure.A p-value < 0.05 was considered statistically significant [21].

## Results

### Global burden of anxiety disorders during perimenopause in different regions

As shown in Table 1, the global burden of ASDRs for anxiety disorders during the perimenopause has increased from 625.51 (95% UI: 429.1–891.09) to 677.15 (95% UI: 469.45–952.72) from 1990 to 2021, showing an upward trend of EAPC 0.081 (95% CI: 0.0043–0.143). With High SDI (EPAC: -0.048; 95% CI: -0.169–0.073),High-middle SDI (EPAC: -0.120; 95% CI: -0.228–0.012), Middle SDI (EPAC: -0.090; 95% CI: -0.257–0.077), East Asia (EPAC: -0.628; 95% CI: -0.865–0.391), High-income Asia Pacific (EPAC: -0.100; 95% CI: -0.176–0.024), High-income North America (EPAC: -0.138; 95% CI: -0.386–0.111), South Asia (EPAC: -0.080; 95% CI: -0.350–0.189) regions showed a declining trend. Other regions showed an upward trend.

**Table 1:** Trends in ASDRs for anxiety disorder in menopausal women from 1990 to 2021

Characteristics	ASDRs(per 100 000)		EAPC((95% CI)
	1990 Rate(95% UI)	2021 Rate(95% UI)	
Global	625.51 (429.10-891.09 )	677.15 (469.45-952.72 )	0.081 (0.004–0.143 )
High SDI	685.58 (471.63-969.51 )	750.26 (519.08-1051.88 )	-0.048 (-0.169-0.073 )
High-middle SDI	634.83 (429.52-905.98 )	674.29 (458.10-951.89 )	-0.12 0(-0.228–0.012 )
Middle SDI	621.29 (426.16-873.74 )	689.08 (474.00-949.74 )	-0.090 (-0.257-0.077 )
Low-middle SDI	540.60 (370.63-770.71 )	605.64 (406.76–857.6 )	0.101 (-0.072-0.274 )
Low SDI	478.29 (317.36-703.01 )	511.44 (337.45-764.02 )	0.027 (-0.062-0.116 )
Andean Latin America	801.3 0(515.84-1213.57 )	941.69 (609.92-1485.64 )	0.187 (0.037–0.337 )
Australasia	662.40 (435.30-1005.79 )	679.33 (416.03-1043.43 )	0.231 (0.072–0.39 )
Caribbean	660.9 0(420.64-1009.47 )	729.85 (466.47-1132.16 )	0.114 (0.031–0.197 )
Central Asia	353.58 (220.17-547.88 )	393.18 (248.30-613.63 )	0.083 (-0.001-0.167 )
Central Europe	597.63 (390.05-887.43 )	670.58 (439.01-994.48 )	0.074 (-0.017-0.165 )
Central Latin America	558.42 (381.44-814.32 )	635.17 (418.57-919.97 )	0.147 (0.032–0.262 )
Central Sub-Saharan Africa	506.15 (314.51-783.71 )	539.36 (332.35-847.93 )	0.113 (0.045–0.182 )
East Asia	606.95 (410.26-864.33 )	617.60 (421.06-862.25 )	-0.628 (-0.865–0.391 )
Eastern Europe	543.62 (366.88-756.99 )	626.07 (428.99–878.8 0)	0.117 (0.011–0.222 )
Eastern Sub-Saharan Africa	521.65 (343.64-781.01 )	573.97 (377.85-849.02 )	0.151 (0.079–0.223 )
High-income Asia Pacific	371.32 (248.85-528.29 )	392.4 (260.83-558.32 )	-0.100 (-0.176–0.024 )
High-income North America	773.45 (539.00-1065.93 )	863.02 (602.23-1185.68 )	-0.138 (-0.386-0.111 )
North Africa and Middle East	664.06 (434.47-984.86 )	738.99 (478.98-1098.43 )	0.320 (0.234–0.407 )
Oceania	646.24 (397.89-1001.26 )	684.63 (414.61-1107.85 )	0.067 (0.024–0.110 )
South Asia	502.80 (343.85-711.07 )	549.42 (375.74-781.76 )	-0.080(-0.350-0.189 )
Southeast Asia	582.29 (392.48-842.68 )	662.71 (438.37-951.99 )	0.183 (0.109–0.257 )
Southern Latin America	751.64 (503.49-1126.59 )	834.41 (522.19-1268.05 )	0.115 (0.014–0.216 )
Southern Sub-Saharan Africa	553.01 (371.36-780.15 )	629.42 (428.25-903.53 )	0.099 (-0.002-0.199 )
Tropical Latin America	968.67 (672.6-1332.29 )	1279.68 (888.75-1777.78 )	0.809 (0.441–1.177 )
Western Europe	827.86 (565.51-1200.16 )	943.49 (645.62-1366.58 )	0.287 (0.194–0.381 )
Western Sub-Saharan Africa	378.53 (251.04-555.32 )	401.46 (263.18-586.36 )	0.167 (0.084–0.25 )

### Global burden of anxiety disorders during perimenopause in various countries

**Fig. 1:**
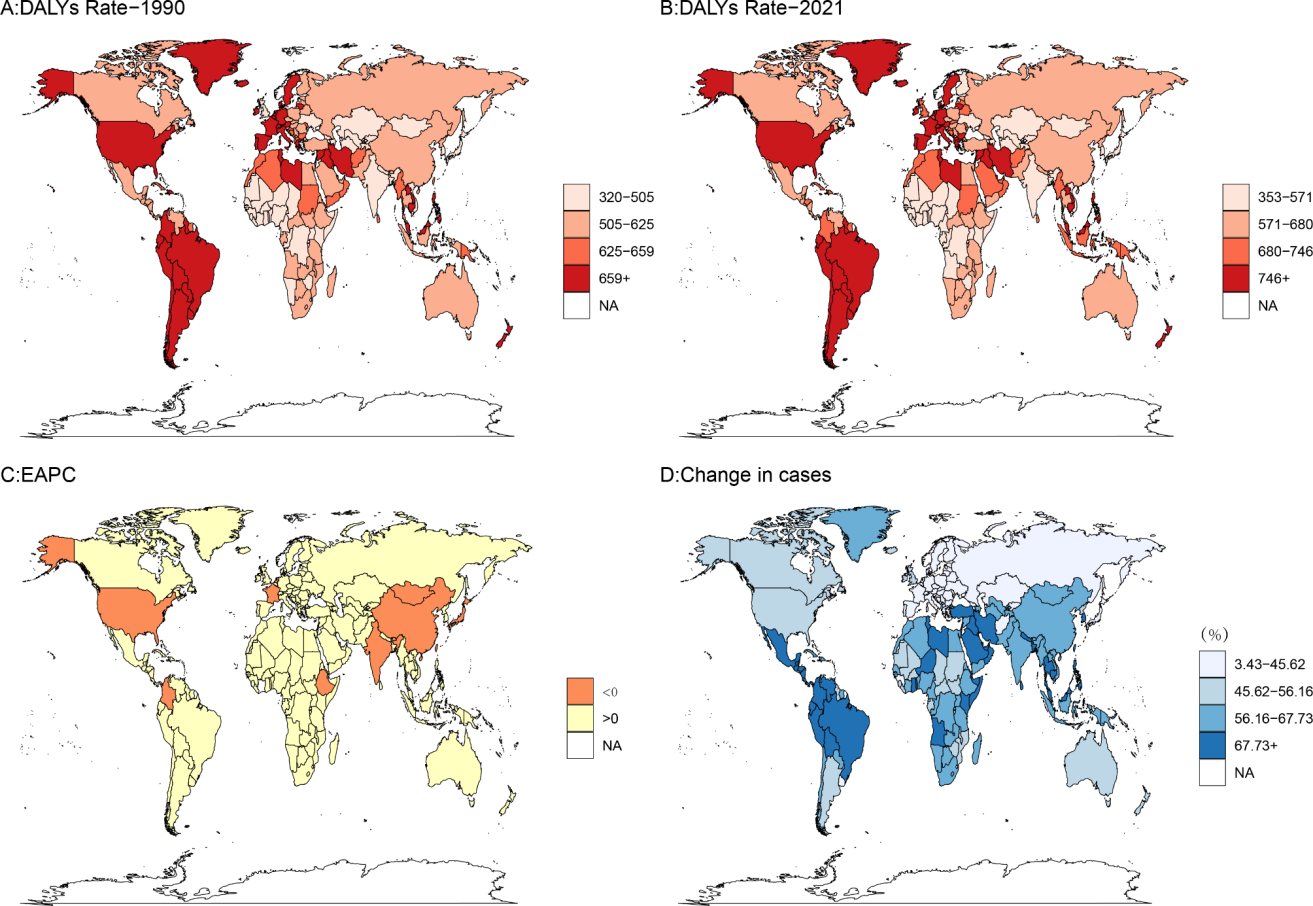
Changes in the burden of anxiety disorders during perimenopause and menopause across various countries

Figure 1 illustrates the global burden of anxiety disorders during perimenopause across 204 countries. The highest disability rates in both 1990 and 2021 were predominantly concentrated in South American and African nations (Fig. 1A and B). From 1990 to 2021, only 12 countries experienced a decline in the disease burden of perimenopausal anxiety disorders (Fig. 1C). However, the total number of disability cases increased in all countries, with 145 countries experiencing an increase of over 50% in incidence rates (Fig. 1D).

### Relationship between ASR and SDI

**Fig. 2:**
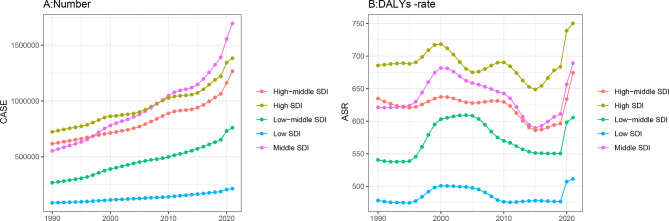
Trends in the burden of anxiety disorders during perimenopause and menopause in different SDI regions from 1990 to 2021

**Fig. 3:**
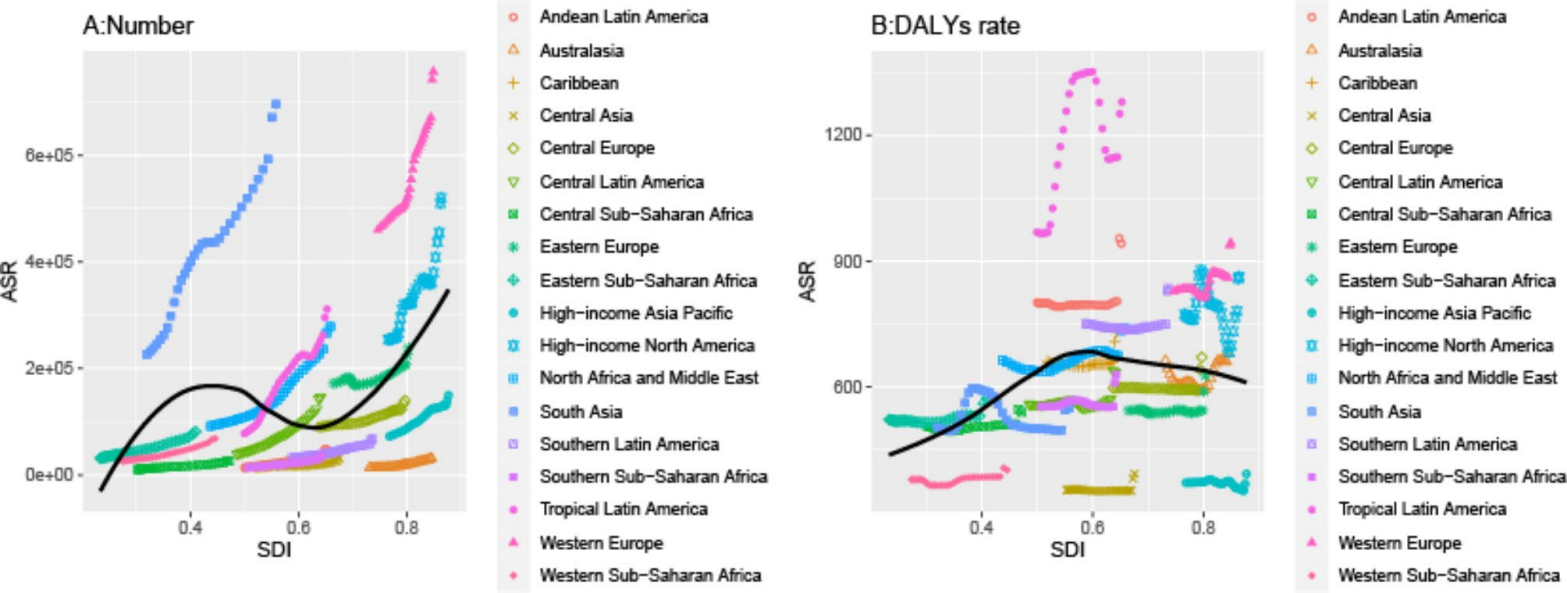
Correlation between the disability rate of anxiety disorders during perimenopause and menopause and SDI in different states

As demonstrated in Fig. 2, the burden and disability associated with anxiety disorders during perimenopause have shown an upward trend across all SDI regions. Between 1990 and 2021, fluctuations in the burden and disability rates were observed (Fig. 2). A nonlinear model was used to explore the correlation between SDI and ASDR by region. The number of disabled individuals increased with rising SDI levels, while the disability rate initially increased and then decreased as SDI rose, peaking at an SDI value of approximately 0.6, followed by a subsequent decline as SDI values continued to rise (Fig. 3).

### Prediction of the global trend in the burden of anxiety disorders during perimenopause, 2022–2035

**Fig. 4:**
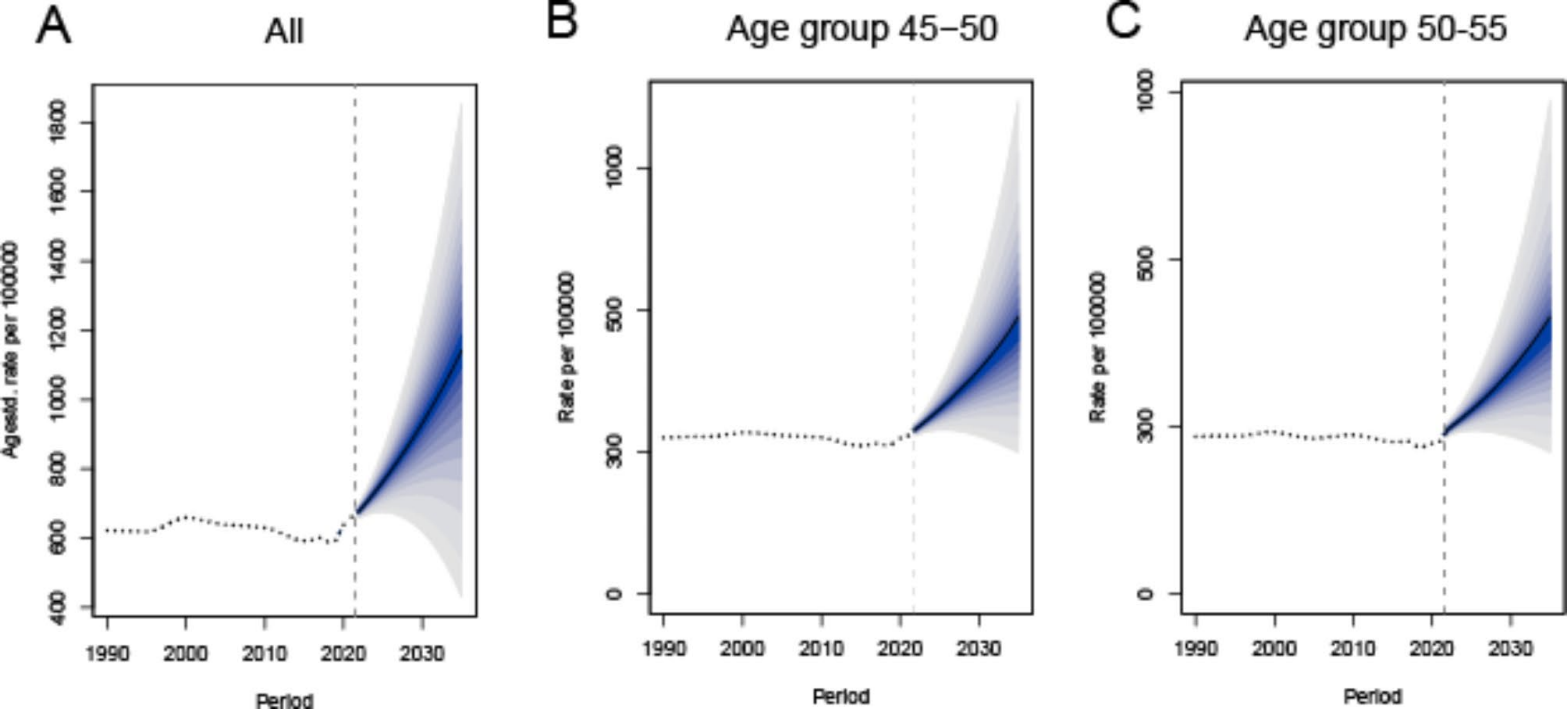
Predicted trends in the burden of anxiety disorders during perimenopause and menopause up to 2035

Figure 4 presents predictions for the global burden of anxiety disorders during perimenopause up to 2035. It is projected that by 2035, the global burden of anxiety disorders during perimenopause will reach 1180.43 per 100,000, reflecting a 40.67% increase compared to 2021 (Fig. 4A). Among these, the age groups 45–50 and 50–55 years are predicted to experience an increase in ASDRs from 2022 to 2035 (Fig. 4A and B).

## Discussion

This study investigates the global trends in the burden of anxiety disorders during perimenopause based on data from the GBD database. The findings suggest that the burden of anxiety disorders in perimenopausal women will remain a significant global concern in the coming years. As such, it is imperative to focus on anxiety disorders during perimenopause by formulating preventive measures and providing effective treatment strategies.

Research has shown that the burden of perimenopausal anxiety disorders exhibits a declining trend in regions with High SDI, High-middle SDI, and Middle SDI. This suggests that there may be regional differences in the burden of perimenopausal anxiety disorders. The reason may be that SDI plays a crucial role in influencing this trend, with factors such as education levels and fertility rates having a direct impact on menopausal anxiety. Studies have also found differences in disability rates across different countries, indicating that perimenopausal anxiety disorders may be related to racial differences. Previous related research has also suggested that the age at menopause and the frequency and severity of symptoms may vary by race.

Anxiety disorders are also influenced by psychological factors. Traditional views consider the changes during the perimenopausal and menopausal stages as a manifestation of “maladaptation.” During this phase, women may lose some dominant roles, such as leaving the workforce, children leaving home, divorce, and the death of loved ones, which can lead to what is known as “empty nest syndrome” and “loss syndrome,” acting as psychological triggers [22]. Related studies indicate that depressive symptoms during the perimenopausal period are more commonly observed in women with introverted personality traits, such as being reclusive, neurotic, and conservative. Research has found that individuals who exhibit higher levels of neural irritability, self-blame, and neuroticism tend to have a higher incidence of anxiety disorders [23].

This study found that as of 2021, the disability rate of peri-menopausal anxiety symptoms is on the rise [0.081 (95% CI: 0.0043–0.143)], which may be related to the current lack of clarity regarding the exact etiology and pathogenesis of peri-menopausal anxiety symptoms. Relevant research suggests that peri-menopausal anxiety may be associated with changes in serotonin, tryptophan hydroxylase, and related factors [24].

Currently, the efficacy of anxiolytic and antidepressant medications such as sertraline and etizolam has been established. These drugs function by upregulating serotonin receptors, enhancing their activity at the synapse, and ultimately alleviating emotional symptoms such as depression [25]. Furthermore, numerous studies have established a link between perimenopausal mood fluctuations and changes in sex hormone levels. The increased prevalence of these emotional disorders in women who have undergone oophorectomy further supports this hypothesis [26]. Some studies have compared morning estrogen levels in perimenopausal women with and without anxiety or depressive symptoms, concluding that women experiencing mood disorders often have lower estrogen levels [27]. Non-pharmacological treatments primarily focus on ensuring sleep quality. Relevant research indicates that peri-menopausal women experience sleep disturbances [28].

In summary, the perimenopausal period is an important stage in the female life cycle and is also a prolonged process. During this stage, women are susceptible to higher levels of anxiety and depression due to various physiological, social, and psychological factors. Therefore, it is essential to implement a combination of interventions, including cognitive guidance, gynecological endocrine management, psychological intervention, and pharmacological treatment. Obstetricians and gynecologists are well-equipped to detect depressive symptoms in the perimenopausal period and can implement standardized detection and management strategies to effectively address these issues [29]. At the same time, improving the socioeconomic level is necessary, particularly raising the SDI. In areas with lower SDI, it is crucial to enhance the management of the physical and mental health of perimenopausal women, leveraging the role of community hospitals to conduct regular high-risk group screenings in the community for early intervention.

This study acknowledges several limitations. The GBD database provides robust epidemiological evidence confirming the anxiety burden among perimenopausal women globally. However, this research is not based on cohort studies, and relevant cohort studies have already been applied in other related disease investigations [30]. Future research should aim to establish relevant cohorts of perimenopausal women and conduct follow-up surveys to identify and understand the specific risk factors contributing to anxiety during this stage.

## Conclusion

The study found that the burden of perimenopausal anxiety is still relatively severe in most parts of the world and is expected to continue rising in the coming years. There is an urgent need for targeted prevention and intervention strategies to alleviate this increasing burden and improve mental health outcomes during the perimenopausal period.

## Data Availability

GBD data used in this work are publicly available. All raw data are available on the GBDwebsite (https://vizhub.healthdata.org/gbd-results/). Further inquiries can be directed to the corresponding author.
